# β-subunit myristoylation functions as an energy sensor by modulating the dynamics of AMP-activated Protein Kinase

**DOI:** 10.1038/srep39417

**Published:** 2016-12-21

**Authors:** Nada Ali, Naomi Ling, Srinath Krishnamurthy, Jonathan S. Oakhill, John W. Scott, David I. Stapleton, Bruce E. Kemp, Ganesh Srinivasan Anand, Paul R. Gooley

**Affiliations:** 1 Department of Biochemistry & Molecular Biology, Bio21 Molecular Science and Biotechnology Institute, 30 Flemington Road, The University of Melbourne, Parkville, Victoria 3010, Australia; 2 Metabolic Signalling Laboratory, St. Vincent’s Institute of Medical Research, University of Melbourne, 41 Victoria Parade, Fitzroy, Victoria 3065, Australia; 3 Department of Biological Sciences, National University of Singapore, 14 Science Dr 4, 117543, Singapore; 4 Department of Protein Chemistry and Metabolism, St. Vincent’s Institute of Medical Research, University of Melbourne, 41 Victoria Parade, Fitzroy, Victoria 3065, Australia; 5 Department of Physiology, The University of Melbourne, Cnr Grattan Street and Royal Parade, Parkville, Victoria 3010, Australia; 6 Mary MacKillop Institute for Health Research, Australian Catholic University, Victoria Parade, Fitzroy, Victoria 3065, Australia

## Abstract

The heterotrimeric AMP-activated protein kinase (AMPK), consisting of α, β and γ subunits, is a stress-sensing enzyme that is activated by phosphorylation of its activation loop in response to increases in cellular AMP. N-terminal myristoylation of the β-subunit has been shown to suppress Thr172 phosphorylation, keeping AMPK in an inactive state. Here we use amide hydrogen-deuterium exchange mass spectrometry (HDX-MS) to investigate the structural and dynamic properties of the mammalian myristoylated and non-myristoylated inactivated AMPK (D139A) in the presence and absence of nucleotides. HDX MS data suggests that the myristoyl group binds near the first helix of the C-terminal lobe of the kinase domain similar to other kinases. Our data, however, also shows that ATP.Mg^2+^ results in a global stabilization of myristoylated, but not non-myristoylated AMPK, and most notably for peptides of the activation loop of the α-kinase domain, the autoinhibitory sequence (AIS) and the βCBM. AMP does not have that effect and HDX measurements for myristoylated and non-myristoylated AMPK in the presence of AMP are similar. These differences in dynamics may account for a reduced basal rate of phosphorylation of Thr172 in myristoylated AMPK in skeletal muscle where endogenous ATP concentrations are very high.

AMPK is an AMP/ATP-sensing enzyme that plays a crucial role in regulating cellular energy metabolism in eukaryotes^1^. Increases in the ratio of AMP/ATP, by processes, such as oxidative stress, glucose deprivation and physical exercise activate AMPK to up-regulate energy-producing processes such as fatty acid oxidation and glycolysis and down-regulate energy-consuming pathways such as fatty acid, triglyceride, and cholesterol synthesis as well as protein synthesis and transcription^2^.

Mammalian AMPK is a heterotrimeric complex consisting of catalytic α- and regulatory β- and γ-subunits^3^,^4^,^5^,^6^,^7^ (Fig. 1). In mammals there are isoforms of each subunit: α1–2^6^, β1–2^8^ and γ1–3^9^ which are differentially expressed in tissues^10^. The α-subunit contains an N-terminal kinase domain (KD) (residues 10–278 in human α1) that includes the essential catalytic loop (135–144) and the activation loop (157–183) which spans the activating phosphorylation site, Thr172^10^, followed by an autoinhibitory sequence (AIS) (294–349), the α-hook (359–365) that has been shown to interact with the γ-subunit^10^,^11^,^12^ and a C-terminal β-subunit interacting domain (β-SID) (395–550)^5^,^6^,^7^,^11^,^12^,^13^. The β-subunit is N-terminal myristoylated at Gly2, which facilitates targeting of AMPK to membranes^14^,^15^. The β-subunit also contains a glycogen targeting carbohydrate-binding module (β-CBM) (residues 74–156, human β1)^16^,^17^,^18^ followed by a C-terminal αγ-subunit binding sequence (αγ-SBS) (184–270)^19^. The γ-subunit is made up of four tandem repeat sequences called cystathionine β synthase (CBS) motifs; CBS1 (residues 56–98, human γ1), CBS2 (137–178), CBS3 (212–251) and CBS4 (285–326) that form two Bateman domains^20^,^21^. While each CBS has an apparent nucleotide-binding site, only three of these sites (1, 3, and 4) are occupied by nucleotide in the crystal structure of mammalian AMPK. CBS1 and CBS3 sites competitively bind to AMP, ATP or ADP whereas CBS4 binds AMP or ATP^21^,^22^,^23^.

Liver kinase β1 (LKB1) and Ca^2+^/Calmodulin-dependent protein kinase β (CaMKKβ) kinases are two of the upstream kinases (AMPKKs) that catalyze the phosphorylation of AMPK at Thr172 of the α-subunit kinase domain, leading to an increase in AMPK activity by several hundred-fold^24^,^25^,^26^,^27^,^28^. The adenine nucleotides (AMP, ADP and ATP) are well characterized as additional non-covalent ligand modulators of AMPK activity. Following phosphorylation of Thr172, AMP-binding to the γ-subunit allosterically increases the activity of the enzyme two to five-fold^9^,^29^ and protects Thr172 from dephosphorylation by protein phosphatases^30^,^31^,^32^.

The N-terminal myristoylation of the β-subunit has a binary function in AMPK regulation. Firstly, it localizes and suppresses activity, as the removal of the myristoyl group by mutation of Gly2 to Ala showed a wide distribution of AMPK into the cytoplasm and a four-fold increase in AMPK activity^14^,^15^. The mechanism of how myristoylation mediates AMPK inhibition is not fully understood. Secondly, it mediates Thr172 phosphorylation in response to the metabolic stress signal from AMP, resulting in maximal AMPK activation. In the absence of myristoylation, this stimulatory effect of AMP is lost, resulting in the abolition of AMPK maximum phosphorylation and activation by AMP^15^. How the myristoyl group modulates activity of AMPK directly and facilitates allosteric interactions with AMP is unclear and represent a major challenge in understanding overall regulation of AMPK function. It seems reasonable that the myristoylation regulatory mechanism involves an interaction between the myristoyl group and an intramolecular binding pocket. Thus far the β-subunit N-terminal myristoyl-group has not been resolved in crystal structures of AMPK. In protein kinase A (PKA) and Abl tyrosine kinase the myristoyl-binding pocket is located in the α-catalytic kinase domain^14^,^15^,^33^,^34^.

In this study, we used amide hydrogen-deuterium exchange mass spectrometry (HDX MS) to map the myristoyl binding pocket as well as structural changes due to myristoylation. In addition, myristoylation-dependent differences in the peptide residues known to have significant roles in interactions with nucleotides were also investigated.

## Results

### Effects of myristoylation on amide hydrogen-deuterium exchange of AMPK

Prior to carrying out the HDX experiments, sequencing of all pepsin digest fragment peptides of non-myristoylated and myristoylated AMPK was carried out as described in materials and methods. A good sequence coverage of 82%, 90% and 91% for α, β and γ subunits respectively of undeuterated non-myristoylated AMPK, and 78%, 86% and 91% for α, β and γ subunits of undeuterated myristoylated AMPK with a large number of overlapping fragments was obtained and analyzed under deuterium exchange conditions (Figs S1 and S2, Tables S1, S2 and S3). For the γ-subunit missing regions were typically one or two residues and therefore all regions believed to be involved in regulation were covered. Sequence coverage missing in other subunits are: α-subunit, 1–21 (only myristoylated), 147–150 and 163–169 (both near the active site), 293–311 (within AIS), 384–416 (linker between the AIS and the β-SID); for the β-subunit, 4–12 (only myristoylated), 85–92 (within the CBM) and 157–168. Two independent HDX experiments were performed each on non-myristoylated and myristoylated AMPK in the presence and absence of nucleotides. Examples of mass changes to the most relevant peptides are reported in Table S4 and show an uncertainty of the measurements to be within ±0.5 deuterons^35^. An uncertainty in measurement ±0.5 deuterons has been identified from across multiple peptides and proteins and has been found to be independent of peptide length, deuterium exchange time and any difference between two states greater than ±0.5 deuterons would imply a change in the physicochemical environment between the two states. Under our experimental conditions and instrumentation with numerous proteins, we do not see a greater uncertainty in measurements of ±0.5 deuterons^36^.

### Mapping interactions of the myristoyl group on AMPK

**Figure 2:**
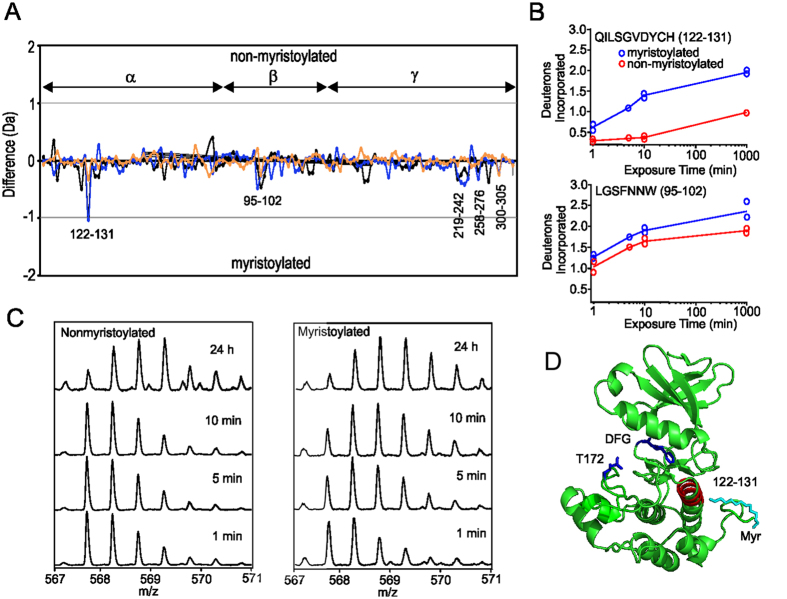
Comparison of deuterium uptake between myristoylated and nonmyristoylated AMPK. (**A**) Difference plot of deuterium uptake for peptides of apo mysritoylated and non-myrisoylated AMPK. Changes in deuteration are shown at three time points: 1 min (orange), 5 min (blue) and 10 min (black). Each point is an average of two separate and independent experiments. On the y-axis a positive difference is indicative of increased deuteration for non-myristoylated AMPK peptides, whereas a negative difference is indicative of increased deuteration for myristoylated AMPK peptides. On the x-axis are individual peptides; assignment of peptide to subunit is shown. (**B**) Time course of deuterium exchange for residues 122–131 of the α-subunit and 95–102 of β-subunit. (**C**) ESI Q-Tof mass spectra of the α-subunit peptide 122–131 at different points of deuteration for non-myristoylated and myristoylated AMPK. (**D**) Location of the α-subunit peptide 122–131 mapped onto the structure of the kinase domain (pdb 4CFH). The position of the phosphorylation site, Thr172, and DFG of the activation loop are shown. In cyan is the position of the myristoyl group after superimposing the structure (pdb 1CMK) of the myristylated kinase domain of cAMP protein kinase, showing that the AMPK peptide 122–131 is near this position.

Considering the general trend of exchange, deuteration in the absence of ligands appears moderately faster for the majority of peptides for myristoylated AMPK compared to non-myristoylated AMPK (Fig. 2). For the β-subunit only the peptide 95–102 of the CBM showed a moderate difference. For the γ-subunit a cluster of peptide fragments showed an increase in HDX for myristoylated AMPK: the region 219–243 of CBS3, 258–276 located between CBS3 and CBS4 and 300–306 from CBS4. Subtractive analysis of overlapping peptides showed that there were no significant differences at spanning residues 119–130, 124–130, 220–227, 300–301 and 305–306 between the apo non-myristoylated/myristoylated proteins, indicating that the most significant differences could be localized to peptides spanning residues 228–243 and 302–304.

The most significant difference between myristoylated and non-myristoylated AMPK for the α-subunit is observed for the peptide 122–131 of the kinase domain, which showed a substantial increase in HDX for myristoylated AMPK (Fig. 2). This peptide showed for non-myristoylated AMPK an incorporation of 0.4 Deuterons over the course of the experiment, whereas for myristoylated AMPK this peptide showed a 1.4 Deuteron increase (Fig. 2B). Subtractive analysis of overlapping peptides show that this increase in deuterium uptake can be localized to Tyr129 which is located at the C-terminal end of the first α-helix in the C-terminal lobe (Fig. 2C). The peptide 112–128, which encompasses all but the last two turns of this helix and therefore excludes this tyrosine, shows minimal HDX (0.48 Deuterons) for both non-myristoylated and myristoylated AMPK suggesting that the helix is well-stabilized by hydrogen bonds. Therefore the significant difference in HDX of the C-terminal end of this helix of non-myristoylated and myristoylated AMPK suggests the location of the myristoyl pocket is near the interface of the DFG motif and the catalytic loop (Fig. 2D), in a similar position as shown by the crystal structure of PKA^33^.

### Mapping effects of AMP on non-myristoylated and myristoylated AMPK

**Figure 3:**
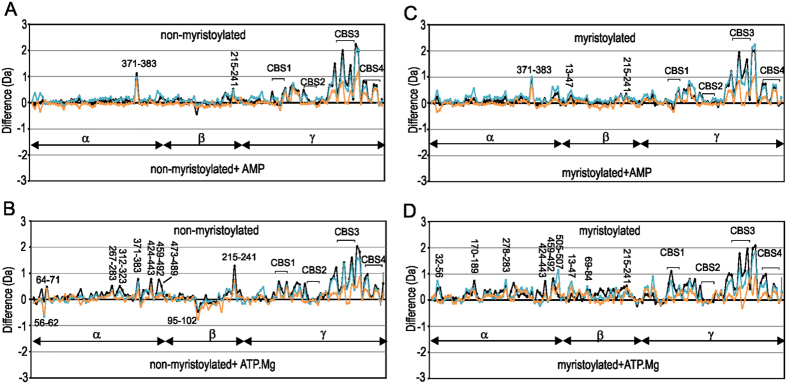
Comparison of deuterium uptake of myristoylated and non-myristoylated AMPK in the presence and absence of the nucleotides AMP and ATP.Mg^2+^. Difference plots of non-myristoylated AMPK with and without (**A**) AMP and (**B**) ATP.Mg^2+^. Difference plots of myristoylated AMPK with and without (**C**) AMP and (**D**) ATP.Mg^2+^. For each plot changes in deuteration are shown at three time points: 1 min (orange), 5 min (blue), 10 min (black). Each point is an average of two separate and independent experiments. On the y-axis a positive difference is indicative of increased deuteration for peptides of the apo-states of AMPK, whereas a negative difference is indicative of increased deuteration for peptides of the nucleotide bound states of AMPK. On the x-axis are individual peptides; assignment of peptide to subunit is shown.

We next set out to monitor the effects of AMP on dynamics of non-myristoylated and myristoylated AMPK. The addition of AMP to both non-myristoylated and myristoylated AMPK has similar effects to each other and is consistent with previously reported results^37^ (Fig. 3A and C). It is noted that experiments were conducted under non-saturating conditions. Assuming the K_d_ for CBS 1 is 2.5 μM^30^ this site would be 96% saturated at 60 μM AMP, whereas CBS3, with a K_d_ of 80 μM, would be 42% saturated. The most significant effects observed are within the γ-subunit: peptides within CBS3 and between CBS3 and CBS4 show the most substantial protection upon binding AMP and to a lesser extent those within CBS4. The increase in protection likely reflects AMP binding to site 3. Peptides within CBS1 show a small degree of increased protection whereas those in CBS2 demonstrate very little protection. However, peptides between CBS1 and CBS2 (108–115 and 116–123) show an increase in protection, and these would reflect binding of AMP to site 1. The marked difference in deuteration for site 3 despite its lower level of saturation with nucleotide perhaps suggests a greater change in dynamics at site 3 and is consistent with previous studies^37^. Outside of the γ-subunit very few differences are observed. The peptide 371–383 of the α-subunit showed protection in the presence of AMP for both proteins. This peptide is C-terminal to the α-hook, although importantly peptides of this latter region showed no differences in HDX. Indeed the peptide 351–366 which encompasses the α-hook gains 3 Deuterons under all conditions. This gain is not surprising as examination of the available structures show the NH groups of the α-hook are exposed and not engaged in hydrogen bonds.

**Figure 4:**
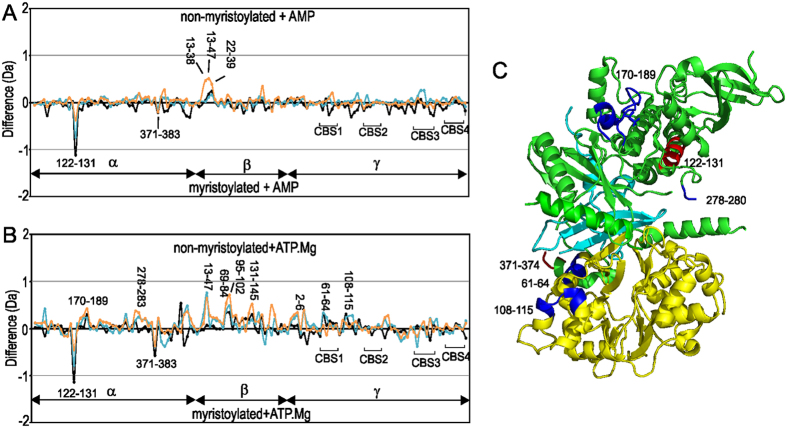
Comparison of deuterium uptake of myristoylated vs non-myristoylated AMPK bound to nucleotides. Difference of myristoylated and non-myristoylated AMPK bound to (**A**) AMP and (**B**) ATP.Mg^2+^. For each plot changes in deuteration are shown at three time points: 1 min (orange), 5 min (blue) and 10 min (black). Each point is an average of two separate and independent experiments. On the y-axis a positive difference is indicative of increased deuteration for peptides of the non-myristoylated AMPK titrated with ATP.Mg^2+^, whereas a negative difference is indicative of increased deuteration for peptides of myristoylated AMPK titrated with ATP.Mg^2+^. On the x-axis are individual peptides; assignment of peptide to subunit is shown. (**C**) Location of peptides that show marked HDX differences in the α- (green) and γ-subunits (yellow) for ATP.Mg^2+^ are mapped onto the structure of AMPK (pdb 4CFH). The αγ-SBS of the β-subunit is in cyan. Significant peptides that show faster HDX for myristoylated AMPK compared to non-myristoylated are mapped on the structure in red and those that show slower HDX in blue. The position of the β-CBM is unknown in this structure, however several peptides of the CBM show slow HDX for myristoylated AMPK.

Comparison of AMP binding to non-myristoylated and myristoylated AMPK highlights several features (Fig. 4A). It is clear that the binding of AMP has the same effects on the γ-subunit of both proteins. Also the peptide 122–131 of the α-subunit, which is a signature of the myristoylation binding pocket, shows similar protection for deuterium exchange in the presence and absence of nucleotide indicating that the addition of AMP has no effect on its HDX profile and suggests that the myristoyl group remains associated with the protein. Our data, however, do not rule out the possibility that AMP-induced myristoyl exposure is suppressed in the absence of an alternative, favourably hydrophobic environment i.e cell membrane, or that the myristoyl group is relocated to a secondary AMPK binding pocket upon AMP binding.

### Mapping effects of ATP.Mg^2+^ binding on non-myristoylated and myristoylated AMPK

The expected K_d_ of ATP.Mg^2+^ for site 1 and site 3 is 18 and 230 μM respectively, therefore we expect under our sample conditions site 1 to be 76% saturated and site 3 to be 21% saturated. The addition of ATP.Mg^2+^ to both non-myristoylated and myristoylated AMPK show broadly similar effects with respect to the γ-subunit as observed for AMP (Fig. 3). Peptides within CBS3 and between CBS3 and CBS4 show substantial protection on binding ATP.Mg^2+^ and to a lesser extent those of CBS1, CBS4 and between CBS1 and CBS2, while CBS2 shows only a small degree of increased protection. Outside of the γ-subunit differences are observed (Figs 3B,D and 4). For non-myristoylated AMPK, ATP.Mg^2+^ showed a marked protection of α-subunit peptides spanning residues 424–443, 459–492 and 473–489 within the β-SID and a reasonable protection on the 312–323 AIS-peptide segment. In addition, within the β-subunit, a decrease in protection of 95–102 within the CBM and a significant protection in peptide 215–241 within the αγ-SBS is observed. Subtractive analysis of αγ-SBS overlapping peptides showed that there were no substantial alterations on the amount of deuterons gained for spanning residues 221–234 and 227–238 indicating that the major differences could be limited to peptides spanning residues 215–221 and 239–241. For myristoylated AMPK, ATP.Mg^2+^ binding has a different effect (Fig. 3D). In comparison to apo myristoylated AMPK there is a general increase in protection for HDX for the bound state, suggesting a globally stabilizing effect (Fig. 3D) and differs from non-myristoylated AMPK bound to ATP.Mg^2+^ (Fig. 4B). Several peptides within the α-subunit kinase domain showed significant decreases in deuterium incorporation in the presence of ATP.Mg^2+^, particularly for the phosphorylation loop that includes Thr172 (170–189) and a connecting loop (278–283) to the AIS (Fig. 3D). To a lesser degree other AIS peptide fragments that span 312–367 showed marked protection in myristoylated AMPK bound to ATP.Mg^2+^, as did the segments spanning the residues 424–507 within the β-SID. For the β-subunit ATP.Mg^+2^ increased protection of several peptides in myristoylated AMPK compared to non-myristoylated AMPK, including 38–40 and 49–54 of the N-terminal region, 69–84, 95–102 and 131–145 of the CBM, and 215–241 of the C-terminal αγ-SBS domain. Similar to AMP binding to either form of the protein, two peptides which show increased deuteration for myristoylated AMPK are the peptide 122–131 of the α-subunit kinase domain, which is the marker peptide for the myristoyl group binding site, and 371–383, a peptide C-terminal to the α-hook (Fig. 4). The pattern of exchange in the former peptide is similar for apo myristoylated AMPK and AMP or ATP.Mg^2+^ bound suggesting the myristoyl group remains bound.

## Discussion

Under conditions of starvation (high AMP and low glucose) the myristoylated β-subunit promotes AMPK association, phosphorylation and activation at cellular membranes, indicating a myristoyl-switch mechanism^15^. This mechanism is proposed to be accompanied by removal of the myristoyl group from the putative binding pocket to interact with the cell membrane^14^,^15^,^33^,^38^. The molecular details of these processes are not well understood. In this study the HDX profiles of myristoylated and non-myristoylated AMPK characterized a peptide, 122 to 131, that suggests the binding site of the myristoyl group is near the C-terminal end of the first α-helix of the C-terminal lobe of the kinase domain (Fig. 2D). This location is in a similar position as shown by the crystal structure of myristoylated cAMP protein kinase and therefore may represent a common binding site for myristoyl groups in kinases^33^. Unlike the kinases Abl and cAMP protein kinase^33^,^34^ the binding pocket of the myristoyl group does not appear to be a deep cavity within the protein core, and in contrast, results in increased deuterium exchange for this region of myristoylated protein suggesting that the addition of the myristoyl group has destabilized this part of the protein. In the presence of AMP and ATP.Mg^2+^, the faster exchange for the α-subunit 122–131 peptide is consistently observed for myristoylated protein (Fig. 4). As we have analysed a kinase dead construct that is not phosphorylated, this result suggests that in the absence of phosphorylation the myristoyl group remains bound. Other than the difference in this peptide, the addition of AMP has little difference on the HDX profiles for myristoylated compared to non-myristoylated AMPK suggesting the overall protein dynamics of these two forms of AMPK are similar in the presence of AMP (Fig. 4A). In contrast, the addition of ATP.Mg^2+^ results in widespread protection of myristoylated compared to non-myistoylated AMPK (Fig. 4B).

There are several interesting similarities and some notable differences between myristoylated and non-myristoylated AMPK in the presence of ATP.Mg^2+^. Firstly, the γ-subunit for both myristoylated and non-myristoylated show similar HDX patterns in the presence of ATP.Mg^2+^. The CBS3 and the connection between CBS3 and CBS4 show the most significant changes on addition of nucleotide for either form of the protein, and our observations are similar to those previously reported^37^. Importantly, these similar effects that we observe for CBS3 of myristoylated and non-myristoylated support the robustness of the methodology and serve as a control for additional comparisons. Several additional small changes of the γ-subunit, especially for peptides encompassing residues 108 to 115 of CBS1, are observed. CBS1 is essentially on the opposite side of the protein to the putative myristoyl-binding pocket (Fig. 4C). Why these peptides show a small increase in protection from exchange for the myristoylated form, however, is not clear.

Secondly, a large number of peptides in the β-subunit show a significant increase in protection for myristoylated AMPK compared to non-myristoylated in the presence of ATP.Mg^2+^ (Fig. 4B). Peptides 69 to 84, 95 to 102 and 131 to 145, are all located in the β-CBM, and all show increased protection from HDX for myristoylated AMPK. These differences are of significant interest as recently the CBM has been shown to be structurally important in the allosteric activation of AMPK by small molecules^12^. In the structure stabilized by the small molecule activator compound 991, the CBM sits on top of the N-terminal lobe of the α-kinase domain to form a hydrophobic drug binding pocket (ADaM site)^39^. In this structure (pdb 4CFF) the peptide 69 to 84 is at the interface of the CBM with the α-kinase domain, but the other peptides are on the surface. However, it is unlikely that the CBM is always located in such a position. A critical residue implicated in this allosteric activation is Ser108 of the β-CBM. This residue has been shown to be cis-autophosphorylated^40^ implying that the CBM needs to reposition near the catalytic site for cis-autophosphorylation of Ser108 to occur. In the kinase dead construct that we have used here, Ser108 will not be phosphorylated and therefore may occupy several positions, including near the substrate/catalytic site of the kinase domain. As HDX of the β-CBM has been slowed for myristoylated AMPK this suggests that myristoylation may have the effect of slowing the mobility and positioning of the CBM, which occurs in the presence of ATP.Mg^2+^ but not AMP.

Thirdly, in the presence of ATP.Mg^2+^ two regions of the α-subunit show increased protection from HDX in the myristoylated form compared to the non-myristoylated; peptide 170 to 189, which includes the phosphorylation site Thr172, and peptide 278 to 283, which is N-terminal to the AIS. The latter peptide, 278 to 283, is on the opposite side of the putative myristoyl-binding pocket and is likely to be near to the myristoyl group and in contrast to the α-subunit peptide 122 to 131 has possibly been stabilized by interacting with the myristoyl group. The peptide 170 to 189, however, is distant from both these regions, but its reduced flexibility and therefore accessibility is consistent with an expected lowering of the basal phosphorylation of Thr172 for myristoylated AMPK^15^.

In conclusion, comparison of the HDX of myristoylated and non-myristoylated kinase dead AMPK suggest a binding site for the myristoyl group similar to other myristoylated kinases. Myristoylation resulted in increased deuteration for the peptide 122 to 131 that marks this binding site, however in the presence of ATP.Mg^2+^ a general protection of deuteration for peptides of the α- and β-subunit were observed, consistent with reduced flexibility of AMPK which is typically expected for myristoyl groups buried in a protein core. Importantly, physiologically AMPK will always be in relatively high concentrations of ATP.Mg^2+^ (ATP, 1 to 10 mM; AMP an order of magnitude lower)^41^. The slowed dynamics we have observed for myristoylated AMPK compared to unmyristoylated in the presence of ATP.Mg^2+^ are likely to be physiologically relevant. A relative increase in AMP levels, and binding to AMPK, would lead to an increase in the flexibility of myristoylated AMPK allowing access to the phosphorylation site and mobility of the β-CBM to occur.

## Methods

### Expression and Purification of Recombinant AMPK Complex

Heterotrimeric human kinase dead AMPK His_6_-α1(D141A)β1γ1 was expressed in *E. coli* strain Rosetta (DE3) using the pET DUET expression system (Novagen) as described previously^40^. Briefly, α1(D141A) and γ1, cloned into pET DUET, were co-expressed with β1, cloned into pET RSF DUET. AMPK stoichiometrically myristoylated on the β subunit residue Gly2 was generated by co-expression with N-myristoyltransferase inserted into pET RSF DUET MCS2 (BglII/XhoI)^15^. Expression cultures were grown in Luria Bertani broth and induced at 16 °C with 0.25 mM isopropyl β-D-thiogalactopyranoside, prior to overnight incubation. Cells were lysed in 50 mM Tris.HCl, pH 7.8, 150 mM NaCl, 10% glycerol, 50 mM imidazole, 2 mM β-mercaptoethanol, 0.1 mM Leupeptin, 0.1 mM AEBSF and 1 mM Benzamidine HCL using a pre-cooled EmulsiFlex-C5 homogenizer (Avestin). AMPK purified using nickel Sepharose and size exclusion chromatography (HiLoad 16/60 Superdex 200 PG). Final storage buffer consisted of 50 mM Tris.HCl, pH 7.8, 150 mM NaCl, 10% glycerol and 2 mM TCEP. All preparations were verified by TOF mass spectrometry.

### HDX MS analysis at peptide resolution

Deuterium exchange was initiated by diluting 2 μl of 30 μM protein, 10-fold with 18 μl deuterated buffer (50 mM Tris.HCl, pH 7.8, 150 mM NaCl, 2 mM TCEP, 10% glycerol, 100% D_2_O) without ligand (AMP or ATP.Mg^2+^) or with 0.6 μl of 2 mM ligand pre-dissolved in 17.4 μl deuterated buffer. The final concentrations of the protein and ligand in the exchange reactions were 3 and 60 μM, respectively. The labelling mixtures were incubated at room temperature and deuterium exchange was carried out for three time points (1, 5 and 10 min), followed by quenching of the reaction, by lowering the pH to 2.5 via addition of 230 μl of pre-chilled 2% trifluoroacetic acid. Quenched samples were digested, desalted and separated online using a Waters ultra-performance liquid chromatography system based on a nanoACQUITY platform as described^36^,^42^. A 250 μl quenched sample was injected into a 2.1 × 30 mm immobilized pepsin column (Porozyme, ABl, Foster City, CA) maintained at 0 °C^43^. The online pepsin proteolysis was carried out for 2.5 min in water containing 0.05% formic acid at a flow rate of 100 μl.min^−1^. The resulting peptic peptides were trapped for 7 min on a 2.1 × 5 mm C18 peptide trap (ACQUITY UPLC BEH C18 VanGuard, 1.7 μm resin, Waters, Milford, MA). The trapped peptides were eluted from the trap column onto a reversed phase Waters ACQUITY UPLC BEH C18 1.7 μm, 1 × 100 mm column held at 0 °C using an 8 to 40% gradient of acetonitrile in 0.1% formic acid at 40 μL.min^−1^ over 10 min. The eluate was directed into a Synapt ESI Q-Tof mass spectrometer (Waters, Manchester, UK). Mass spectra were acquired in MS^E^ mode, a nonbiased, nonselective CID method^44^. Peptide sequence identifications were performed from undeuterated samples using ProteinLynx Global Server 2.5 (beta test version) (Waters, Milford, MA) and searched against the sequence of AMPK with no protease specified. Peptide deuterium exchange was calculated using DynamX 2.0 software (Waters, Milford, MA). No adjustment was made for deuterium back-exchange during analysis and therefore all results are reported in absolute exchange terms. The average back exchange we have observed in our HDX MS system is about 30%^45^.

## Additional Information

**How to cite this article**: Ali, N. *et al*. β-subunit myristoylation functions as an energy sensor by modulating the dynamics of AMP-activated Protein Kinase. *Sci. Rep.* **6**, 39417; doi: 10.1038/srep39417 (2016).

**Publisher's note:** Springer Nature remains neutral with regard to jurisdictional claims in published maps and institutional affiliations.

## Supplementary Material

Supplementary Information

## Figures and Tables

**Figure 1:**
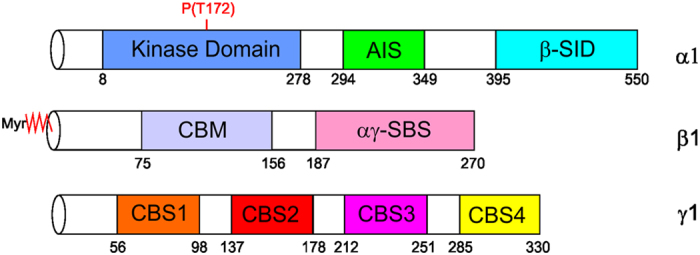
Domain organization of the subunits of AMPK. The domain regions are annotated with residue numbering according to the α1, β1 and γ1 isoforms of mammalian AMPK. The α-subunit consists of an N-terminal kinase domain, an autoinhibitory sequence (AIS) and a β-subunit interacting domain. The β-subunit consists of an N-terminal myristoyl group, carbohydrate binding module (CBM) and C-terminal αγ-subunit binding sequence (αγ-SBS). The γ-subunit consists of four cystathionine β-synthase (CBS) domains.
