# Supplementary material for: β-subunit myristoylation functions as an energy sensor by modulating the dynamics of AMP-activated Protein Kinase
Source: Sci Rep. 2016 Dec 21;6:39417. doi: 10.1038/srep39417 (PMC5175161; doi:10.1038/srep39417)
Supplement: Supplementary Information [file srep39417-s1.pdf]

β-subunit myristoylation functions as an energy sensor by modulating the dynamics of AMP-activated Protein Kinase.

Nada Ali[a], Naomi Ling[b], Srinath Krishnamurthy[c], Jonathan S. Oakhill[b], John W. Scott[d], David I. Stapleton[e], Bruce E. Kemp[d.f], Ganesh S. Anand[c], Paul R. Gooley[a]

(A)

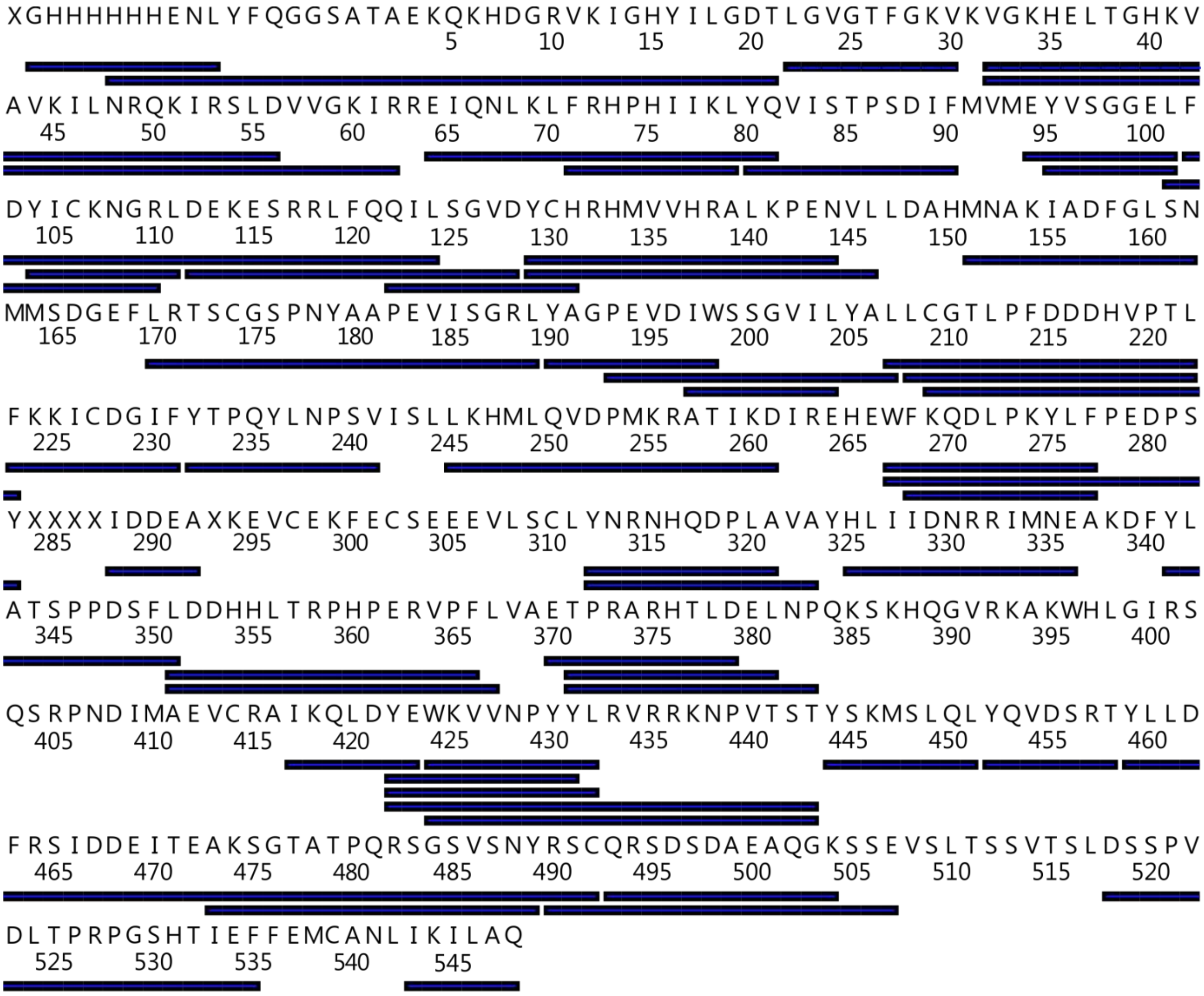
XGHHHHHHENLYFQGGSATAEKQKHDGRVKIGHYILGDTLGVGTFGKVKVGKHELTGHKV
5 10 15 20 25 30 35 40
AVKILNRQKIRSLDVVGKIRREIQNLKLFRHPHIIKLYQVISTPSDIFMVMEYVSGGELF
45 50 55 60 65 70 75 80 85 90 95 100
DYICKNGRLDEKESRRLFQQILSGVDYCHRHMVVHRALKPENVLLDAHMNAKIADFGLSN
105 110 115 120 125 130 135 140 145 150 155 160
MMSDGEFLRTSCGSPNYAAPEVISGRLYAGPEVDIWSSGVILYALLCGTLPFDDDHVPTL
165 170 175 180 185 190 195 200 205 210 215 220
FKKICDGIFYTPQYLNPSVISLLKHMLQVDPMKRATIKDIREHEWFKQDLPKYLFPEDPS
225 230 235 240 245 250 255 260 265 270 275 280
YXXXXIDDEAXKEVCEKFECSEEEVLSCLYNRNHQDPLAVAYHLIIDNRRIMNEAKDFYL
285 290 295 300 305 310 315 320 325 330 335 340
ATSPPDSFLDDHHLTRPHPERVPFLVAETPRARHTLDELNPQKSKHQGVRKAKWHLGIRS
345 350 355 360 365 370 375 380 385 390 395 400
QSRPNDIMAEVCRAIKQLDYEWKVVNPYYLRVRRKNPVTSTYSKMSLQLYQVDSRTYLLD
405 410 415 420 425 430 435 440 445 450 455 460
FRSIDDEITEAKSGTATPQRSGSVSNYRSCQRSDSDAEAQGKSSEVSLTSSVTSLDSSPV
465 470 475 480 485 490 495 500 505 510 515 520
DLTPRPGSHTIEFFEMCANLIKILAQ
525 530 535 540 545

(B)

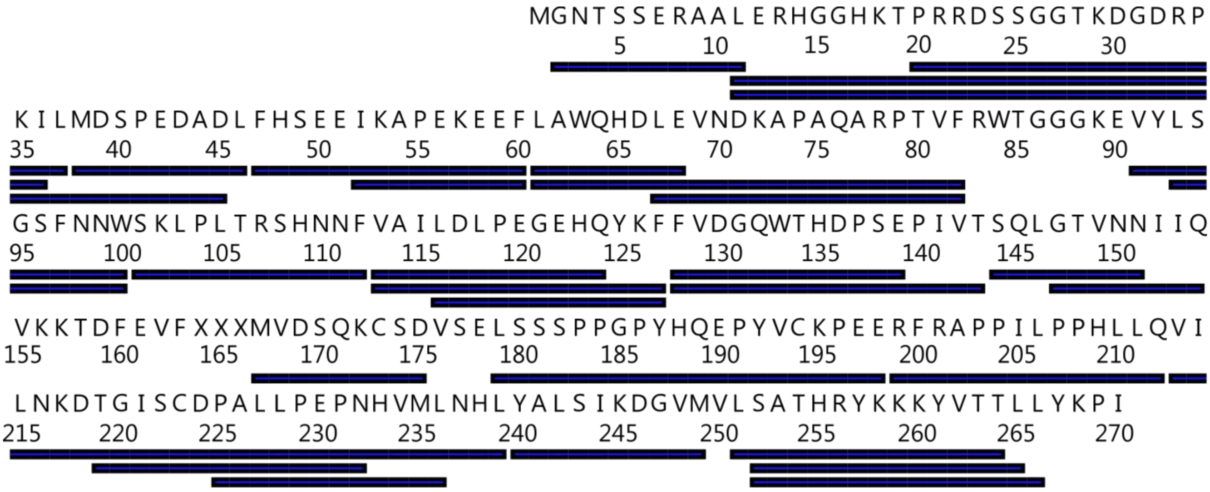
MGNTSSERAALERHGGHKTPRRDSSGGTKDGDRP
5 10 15 20 25 30
KILMDSPEDADLFHSEEIKAPEKEEFLAWQHDLEVNDKAPAQARPTVFRWTGGGKEVYLS
35 40 45 50 55 60 65 70 75 80 85 90
GSFNNWSKLPLTRSHNNFVAILDLPEGEHQYKFFVDGQWTHDPSEPIVTSQLGTVNNIIQ
95 100 105 110 115 120 125 130 135 140 145 150
VKKTDFEVFXXXMVDSQKCSDVSELSSSPPGPYHQEPYVCKPEERFRAPPILPPHLLQVI
155 160 165 170 175 180 185 190 195 200 205 210
LNKDTGISCDPALLPEPNHVMLNHLYALSIKDGVMVLSATHRYKKKYVTTLLYKPI
215 220 225 230 235 240 245 250 255 260 265 270

(C)

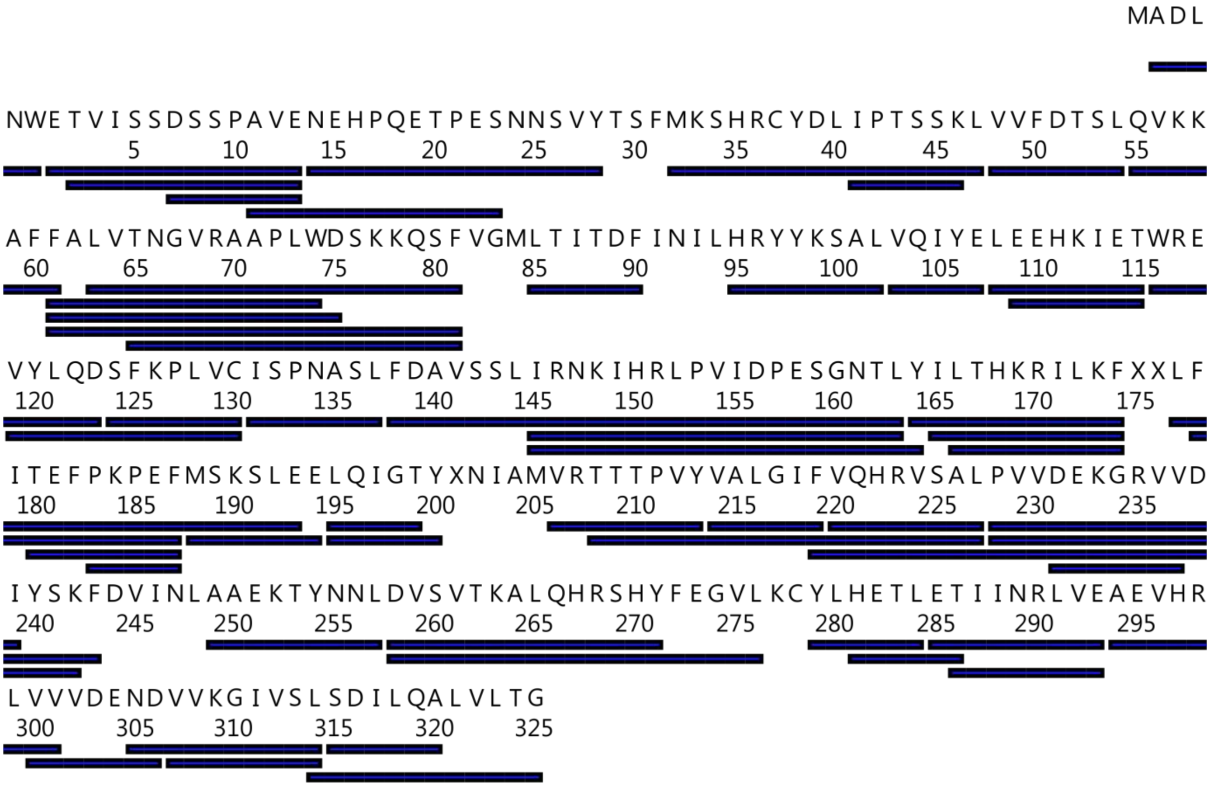

Figure S1: Pepsin digestion coverage map of deuterated nonmyristoylated AMPK. Each blue bar beneath the sequence represents an identified AMPK peptic peptide that provided the present HDX data. Peptide coverage 82%, 90% and 91% of the primary sequence of (A) α-subunit, (B) β-subunit and (C) γ-subunit, respectively. The map was generated with the DynamX 2.0 software.

(A)

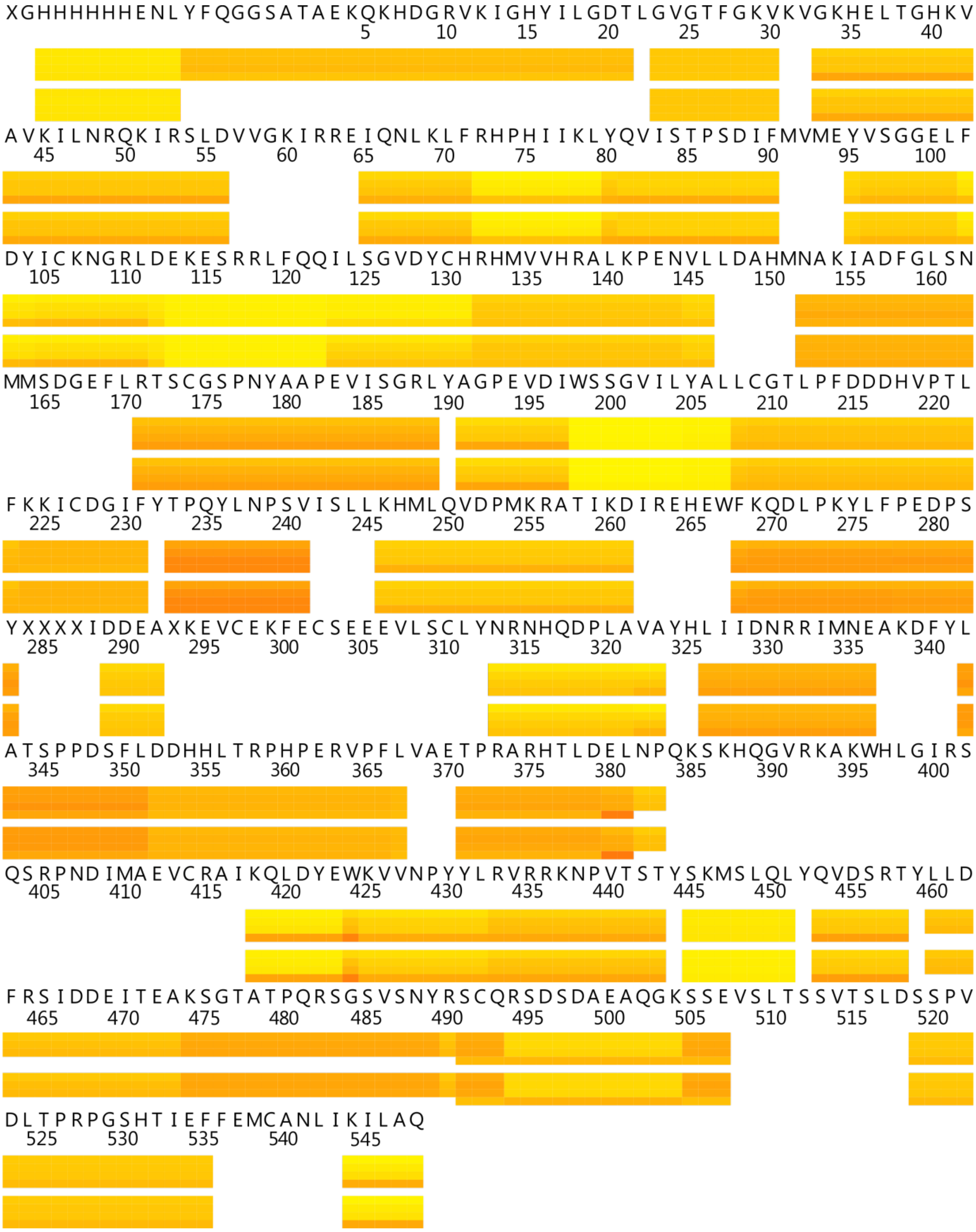
XGHHHHHHENLYFQGGSATAEKQKHDGRVKIGHYILGDTLGVGTFGKVKVGKHELTGHKV
5 10 15 20 25 30 35 40
AVKILNRQKIRSLDVVGKIRREIQNLKLFRHPHIIKLYQVISTPSDIFMVMEYVSGGELF
45 50 55 60 65 70 75 80 85 90 95 100
DYICKNGRLDEKESRRLFQQILSGVDYCHRHMVVHRALKPENVLLDAHMNAKIADFGLSN
105 110 115 120 125 130 135 140 145 150 155 160
MMSDGEFLRTSCGSPNYAAPEVISGRLYAGPEVDIWSSGVILYALLCGTLPFDDDHVPTL
165 170 175 180 185 190 195 200 205 210 215 220
FKKICDGIFYTPQYLNPSVISLLKHMLQVDPMKRATIKDIREHEWFKQDLPKYLFPEDPS
225 230 235 240 245 250 255 260 265 270 275 280
YXXXXIDDEAXKEVCEKFECSEEEVLSCLYNRNHQDPLAVAYHLIIDNRRIMNEAKDFYL
285 290 295 300 305 310 315 320 325 330 335 340
ATSPPDSFLDDHHLTRPHPERVPFLVAETPRARHTLDELNPQKSKHQGVRKAKWHLGIRS
345 350 355 360 365 370 375 380 385 390 395 400
QSRPNDIMAEVCRAIKQLDYEWKVVNPYYLRVRRKNPVTSTYSKMSLQLYQVDSRTYLLD
405 410 415 420 425 430 435 440 445 450 455 460
FRSIDDEITEAKSGTATPQRSGSVSNYRSCQRSDSDAEAQGKSSEVSLTSSVTSLDSSPV
465 470 475 480 485 490 495 500 505 510 515 520
DLTPRPGSHTIEFFEMCANLIKILAQ
525 530 535 540 545

(B)

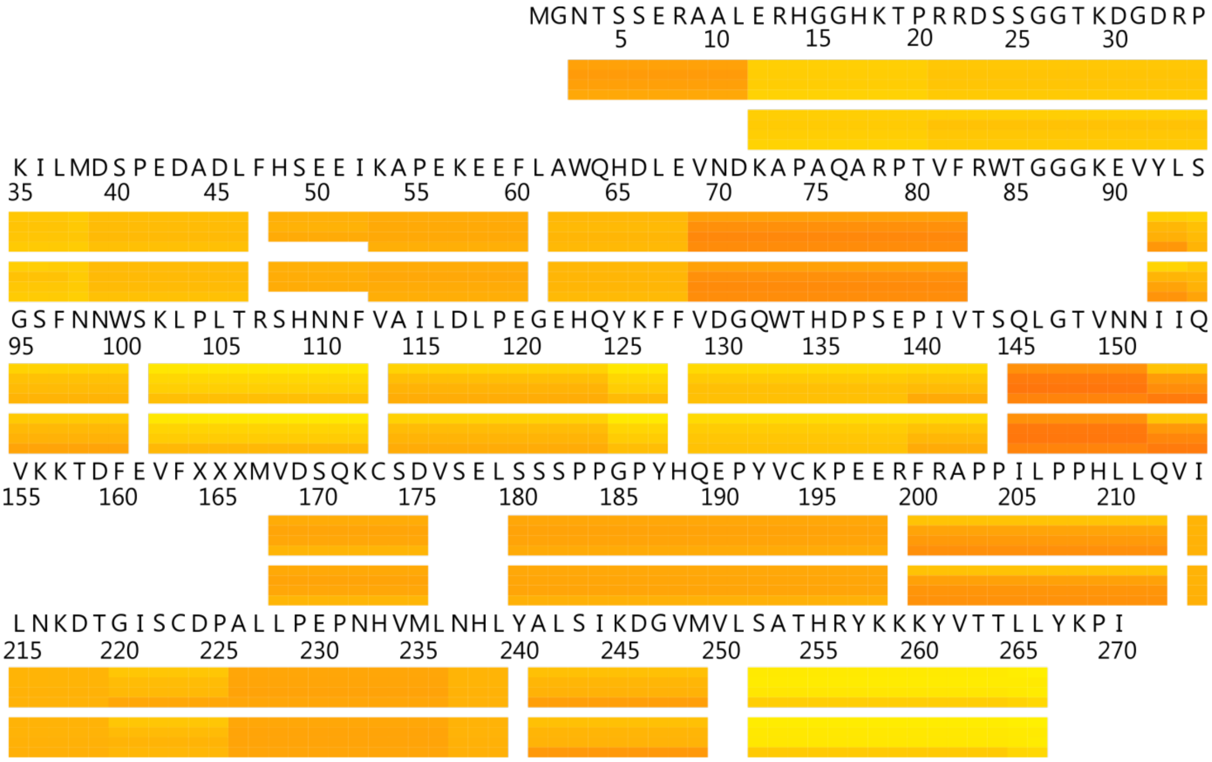
MGNTSSERAALERHGGHKTPRRDSSGGTKDGDRP
5 10 15 20 25 30
KILMDSPEDADLFHSEEIKAPEKEEFLAWQHDLEVNDKAPAQARPTVFRWTGGGKEVYLS
35 40 45 50 55 60 65 70 75 80 85 90
GSFNNWSKLPLTRSHNNFVAILDLPEGEHQYKFFVDGQWTHDPSEPIVTSQLGTVNNIIQ
95 100 105 110 115 120 125 130 135 140 145 150
VKKTDFEVFXXXMVDSQKCSDVSELSSSPPGPYHQEPYVCKPEERFRAPPILPPHLLQVI
155 160 165 170 175 180 185 190 195 200 205 210
LNKDTGISCDPALLPEPNHVMLNHLYALSIKDGVMVLSATHRYKKKYVTTLLYKPI
215 220 225 230 235 240 245 250 255 260 265 270

(C)

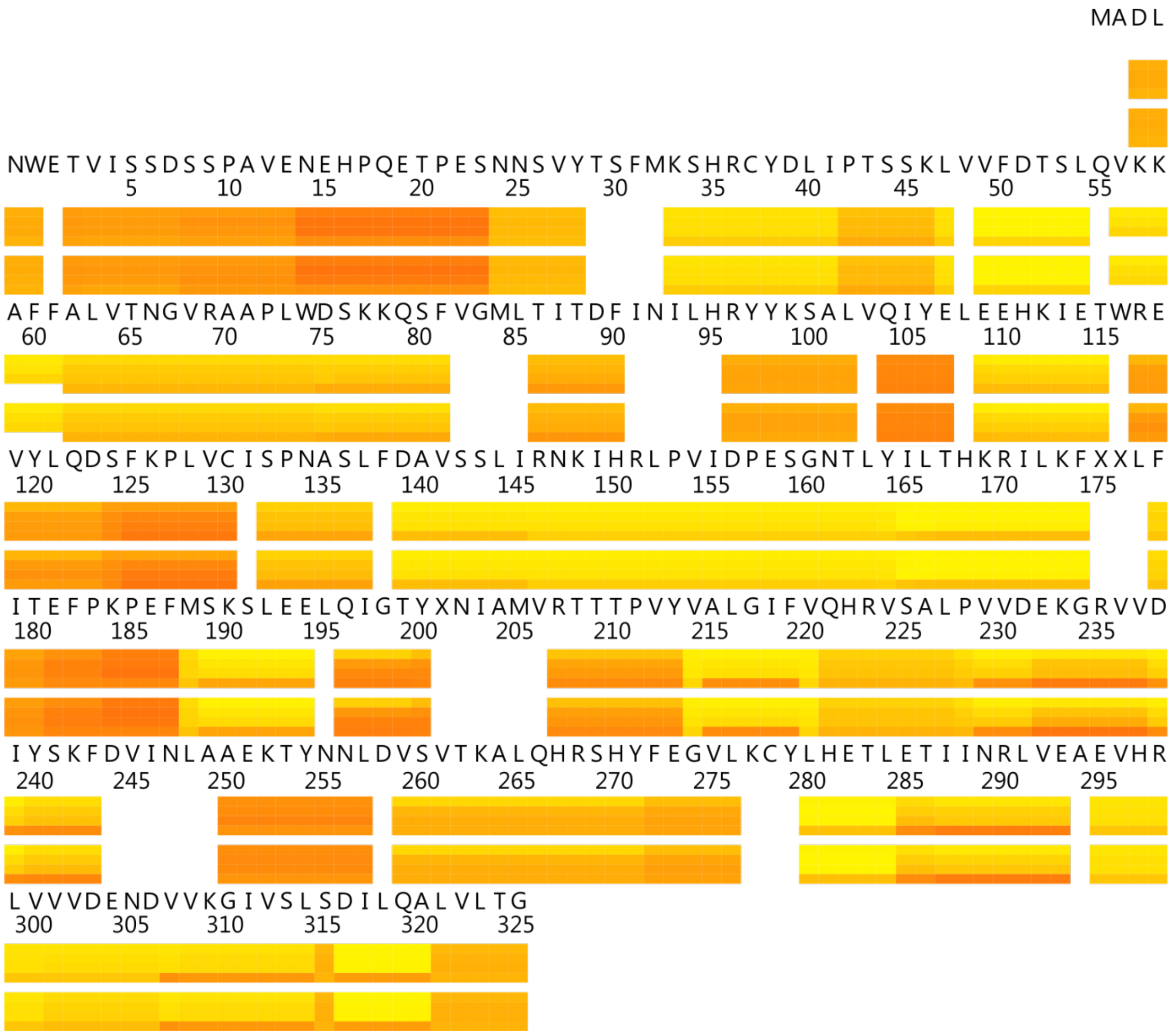

Figure S2: Schematic representation of the relative deuterium incorporation in each identified peptic peptide of apo non-myristoylated and myristoylated AMPK; (A) α-subunit, (B) β-subunit and (C) γ-subunit, at different time point. The colored bars beneath the sequence represent, in descending order, deuterium incorporation at 1 min, 5 min, 10 min, and 24 h. The bar colors range from light yellow to dark orange. The intensity of color indicates the increasing deuterium incorporation; light yellow bar displays the lowest level of deuterium incorporation where dark orange bar displays the highest level of deuterium incorporation. The above map was generated with the DynamX 2.0 software. Labeled residues show differences in deuterium uptake between apo myr (top bars) and nonmyr (bottom bars) AMPK.

Table S1: Sequence identification of alpha subunit of deuterated AMPK. A total of 54 pepsin digest fragments were analyzed. These peptides spanned approximately 82% and 78% of the primary sequence of nonmyr and myr AMPK Alpha subunit, respectively.

Residue Number	Peptide	m/z and Charge
	GHHHHHHENL[a]	627.79 (2)
**1--21**	HHHENLYFQGGSA[a]/TAEKQKHDGRVKIGHYILGDT[b]	641.50 (6)
**22--30**	LGVGTFGKV	439.25 (2)
**32--56**	VGKHELTGHKVAVKILNRQKIRSLD	710.67 (4)
**32--62**	VGKHELTGHKVAVKILNRQKIRSLDVVGKIR	873.88 (4)
**64-81**	EIQNLKLFRHPHIIKLYQ	573.33 (4)
**71--79**	FRHPHIIKL	580.86 (2)
**80--90**	YQVISTPSDIF	1269.64 (1)
**94--101**	EYVSGGEL	853.39 (1)
**95--101**	YVSGGEL	724.35 (1)
**101--111**	LFDYICKNGR	410.20 (3)
**102-124**	FDYICKNGRLDEKESRRLFQQIL	957.84 (3)
**104-111**	YICKNGRL	483.76 (2)
**112-128**	DEKESRRLFQQILSGVD	1010.53 (2)
**122-131**	QILSGVDYCH	567.76 (2)
**129-144**	YCHRHMVVHRALKPEN	995.49 (2)
**129-146**	YCHRHMVVHRALKPENVL	734.72 (3)
**151-162**	MNAKIADFGLSN	640.83 (2)
**170-189**	LRTSCGSPNYAAPEVISGRL	697.69 (3)
**190-198**	YAGPEVDIW	1049.5 (1)
**193-207**	PEVDIWSSGVILYAL	831.44 (2)
**197-204**	IWSSGVIL	874.5 (1)
**207-222**	LLCGTLPFDDDHVPTL	878.44 (2)
**208-222**	LCGTLPFDDDHVPTL	821.9 (2)
**209-223**	CGTLPFDDDHVPTLF	838.89 (2)
**223-231**	FKKICDGIF	535.79 (2)
**232-241**	YTPQYLNPSV	1181.58 (1)
**245-261**	LKHMLQVDPMKRATIKD	1012.55 (2)
**267-277**	WFKQDLPKYLF	742.90 (2)
**267-283**	WFKQDLPKYLFPEDPSY	1087.03 (2)
**268-277**	FKQDLPKYLF	649.86 (2)
**288-292**	IDDEA	562.23 (1)
**312-321**	YNRNHQDPLA	1227.58 (1)
**312-323**	YNRNHQDPLAVA	699.35 (2)
**325-336**	HLIIDNRRIMNE	762.41 (2)
**341-351**	YLATSPPDSFL	1210.6 (1)
**351-366**	LDDHHLTRPHPERVPF	983.5 (2)
**351-367**	LDDHHLTRPHPERVPFL	1040.05 (2)
**370-379**	ETPRARHTLD	598.3 (2)
**371-381**	TPRARHTLDEL	654.86 (2)
**371-383**	TPRARHTLDELNP	380.7 (4)

**417-423**	IKQLDYE	908.47 (1)
**422-431**	YEWKVVNPYY	680.8 (2)
**422-432**	YEWKVVNPYYL	737.38 (2)
**424-432**	WKVVNPYYL	591.32 (2)
**424-443**	WKVVNPYYLRVRRKNPVTST	1238.69 (2)
**444-451**	YSKMSLQL	485.25 (2)
**452-458**	YQVDSRT	868.42 (1)
**459-492**	YLLDFRSIDDEITEAKSGTATPQRSGSVSNYRSC	1256.31 (3)
**473-489**	AKSGTATPQRSGSVSNY	855.92 (2)
**490-507**	RSCQRSDSDAEAQGKSSE	970.93 (2)
**493-504**	QRSDSDAEAQGK	646.31 (2)
**518-535**	DSSPVDLTPRPGSHTIEF	977.98 (2)
**543-548**	IKILAQ	685.46 (1)

[a] His-tag sequence was not included in numeracy as it does not exist in the crystal structure of mammalian AMPK. [b] missing in mass spectra of myristoylated AMPK.

Table S2: Sequence identification of Beta subunit of deuterated AMPK. A total of 29 pepsin digest fragments were analyzed. These peptides spanned approximately 90% and 86% of the primary sequence of nonmyr and myr AMPK beta subunit, respectively.

Residue Number	Peptide	m/z and Charge
4---13	GNTSSERAAL[a]	1005.497 (1)
13-38	LERHGGHKTPRRDSSGGTKDGDRPKI	572.3 (5)
13-47	LERHGGHKTPRRDSSGGTKDGDRPKILMDSPEDAD	766.98 (5)
22-39	PRRDSSGGTKDGDRPKIL	978.02 (2)
40-48	MDSPEDADL	992.4 (1)
49-62	FHSEEIKAPEKEEF	860.41 (2)
54-62	IKAPEKEEF	545.79 (2)
63-70	LAWQHDLE	506.24 (2)
69-84	LEVNDKAPAQARPTVF	585.99 (3)
93-102	VYLSGSFNNW	1186.55 (1)
95-102	LSGSFNNW	924.42 (1)
103-114	SKLPLTRSHNNF	707.38 (2)
115-126	VAILDLPEGEHQ	660.85 (2)
115-129	VAILDLPEGEHQYKF	586.97 (3)
119-129	DLPEGEHQYKF	681.8 (2)
130-141	FVDGQWTHDPSE	709.31 (2)
130-145	FVDGQWTHDPSEPIVT	914.44 (2)
146-153	SQLGTVNN	832.41 (1)
149-156	GTVNNIIQ	858.46 (1)
169-177	MVDSQKCSD	1012.41 (1)
181-200	LSSSPPGPYHQEPYVCKPEE	1122.5 (2)
201-214	RFRAPPILPPHLLQ	552.33 (3)
215-241	VILNKDTGISCDPALLPEPNHVMLNHL	985.18 (3)
221-234	TGISCDPALLPEPN	713.85 (2)
227-238	PALLPEPNHVML	665.86 (2)
242-251	YALSIKDGVM	548.79 (2)
253-266	LSATHRYKKKYVTT	848.48 (2)
254-267	SATHRYKKKYVTTL	565.99 (3)
254-268	SATHRYKKKYVTTLL	905.02 (2)

[a] missing in mass spectra of myristoylated AMPK.

Table S3: Sequence identification of Gamma subunit of deuterated AMPK. A total of 54 pepsin digest fragments were analyzed. These peptides spanned approximately 91% of the primary sequence of both nonmyr and myr AMPK gamma subunit.

Residue Number	Peptide	m/z and Charge
	ADLNW[a]	618.32 (1)
1--13	ETVISSDSSPAVE	1320.62 (1)
2--13	TVISSDSSPAVE	1191.58 (1)
7--13	DSSPAVE	704.31 (1)
11--23	AVENEHPQETPES	733.82 (2)
14--28	NEHPQETPESNNSVY	872.87 (2)
32--47	MKSHRCYDLIPTSSKL	939.98 (2)
41-46	IPTSSK	316.68 (2)
48-54	VVFDTSL	780.14 (1)
55-61	QVKKAFF	867.5 (1)
61-74	FALVTNGVRAAPLW	757.92 (2)
61-75	FALVTNGVRAAPLWD	815.44 (2)
65-81	TNGVRAAPLWDSKKQSF	635.67 (3)
85-90	LTITDF	709.37 (1)
95-102	HRYYKSAL	519.28 (2)
103-107	VQIYE	651.35 (1)
108-115	LEEHKIET	499.76 (2)
109-115	EEHKIET	885.44 (1)
116-123	WREVYLQD	1108.55 (1)
119-130	VYLQDSFKPLVC	706.37 (2)
124-130	SFKPLVC	793.43 (1)
131-137	ISPNASL	701.39 (1)
138-163	FDAVSSLIRNKIHRLPVIDPESGNTL	964.5 (3)
145-163	IRNKIHRLPVIDPESGNTL	1086.61(2)
164-174	YILTHKRILKF	477.96 (3)
165-174	ILTHKRILKF	423.61 (3)
166-174	LTHKRILKF	578.37 (2)
177-193	LFITEFPKPEFMSKSLE	511.52 (4)
178-187	FITEFPKPEF	627.82 (2)
180-187	TEFPKPEF	994.49 (1)
183-187	PKPEF	309.17 (2)
188-194	MSKSLEE	823.38 (1)
195-199	LQIGT	531.31 (1)
195-200	LQIGTY	694.382 (1)
206-213	VRTTTPVY	936.51 (1)
214-219	VALGIF	619.38 (1)
219-242	FVQHRVSALPVVDEKGRVVDIYSK	914.5 (3)
220-227	VQHRVSAL	909.52 (1)
228-239	PVVDEKGRVVDI	663.38 (2)
228-243	PVVDEKGRVVDIYSKF	926.02(2)
231-237	DEKGRVV	401.72 (2)

248-257	AAEKTYNNL	1023.52 (1)
258-271	DVSVTKALQHRSHY	820.93 (2)
258-276	DVSVTKALQHRSHYFEGVL	729.38 (3)
279-284	YLHETL	388.19 (2)
281-286	HETLET	729.34 (1)
285-293	ETIINRLVE	543.81 (2)
286-293	TIINRLVE	957.57 (1)
294-301	AEVHRLVV	461.77 (2)
300-306	VVVDEND	789.36 (1)
305-314	NDVVKGIVSL	1043.61 (1)
307-314	VVKGIVSL	814.54 (1)
314-325	LSDILQALVLTG	415.24 (3)
315-320	SDILQA	646.34 (1)

[a] Linker sequence was not included in numeracy as it does not exist in the crystal structure of mammalian AMPK

Table S4. Summary of $^{1}H/^{2}H$ exchange data for myristoylated and non-myristoylated AMPK[a].

Deuterium exchange (10 min)						
	Non-myristoylated			Myristoylated		
Peptide (α-subunit)	Apo	AMP	$ATP.Mg^{2+}$	Apo	AMP	$ATP.Mg^{2+}$
122-131	0.38 ± 0.08	0.32 ± 0.00	0.21 ± 0.00	1.40 ± 0.04	1.43 ± 0.01	1.35 ± 0.05
170-189	5.16 ± 0.06	5.02 ± 0.01	4.86 ± 0.00	5.29 ± 0.14	5.09 ± 0.06	4.56 ± 0.16
267-283	4.95 ± 0.00	4.45 ± 0.04	4.18 ± 0.06	4.77 ± 0.22	4.44 ± 0.06	3.97 ± 0.07
268-277	2.94 ± 0.15	2.94 ± 0.01	2.74 ± 0.00	3.00 ± 0.07	2.89 ± 0.03	2.68 ± 0.05
371-383	2.14 ± 0.09	1.04 ± 0.04	1.38 ± 0.00	2.23 ± 0.04	1.22 ± 0.01	1.95 ± 0.06
Peptide (β-subunit)	Apo	AMP	$ATP.Mg^{2+}$	Apo	AMP	$ATP.Mg^{2+}$
13-47	6.05 ± 0.00	5.38 ± 0.09	5.24 ± 0.22	5.45 ± 0.10	5.20 ± 0.17	5.05 ± 0.25
69-84	5.83 ± 0.00	5.57 ± 0.00	5.61 ± 0.00	5.68 ± 0.13	5.53 ± 0.05	5.27 ± 0.14
93-102	2.55 ± 0.03	2.97 ± 0.04	3.01 ± 0.00	3.03 ± 0.15	3.05 ± 0.15	2.67 ± 0.11
131-145	3.12 ± 0.06	3.23 ± 0.01	3.24 ± 0.00	3.35 ± 0.14	3.41 ± 0.03	2.94 ± 0.01
Peptide (γ-subunit)	Apo	AMP	$ATP.Mg^{2+}$	Apo	AMP	$ATP.Mg^{2+}$
2-13	3.60 ± 0.27	3.61 ± 0.01	3.55 ± 0.00	3.69 ± 0.06	3.52 ± 0.08	3.48 ± 0.06
61-75	2.29 ± 0.18	2.34 ± 0.08	1.61 ± 0.00	2.38 ± 0.06	2.27 ± 0.12	1.55 ± 0.03
108-115	1.13 ± 0.03	0.58 ± 0.04	0.89 ± 0.04	1.16 ± 0.6	0.67 ± 0.05	0.58 ± 0.02

[a] Average difference in incorporated deueterons at the 10 minute timepoint for the two independent experiments ± S.D.
